# Converging circuits between pain and depression: the ventral tegmental area as a therapeutic hub

**DOI:** 10.3389/fphar.2023.1278023

**Published:** 2023-10-02

**Authors:** Montse Flores-García, Arianna Rizzo, Maria Zelai Garçon-Poca, Víctor Fernández-Dueñas, Jordi Bonaventura

**Affiliations:** ^1^ Unitat de Farmacologia, Facultat de Medicina i Ciències de la Salut, Institut de Neurociències, Universitat de Barcelona, L’Hospitalet de Llobregat, Catalonia, Spain; ^2^ Neuropharmacology and Pain Group, Neuroscience Program, IDIBELL-Institut d’Investigació Biomèdica de Bellvitge, L’Hospitalet de Llobregat, Catalonia, Spain

**Keywords:** ventral tegmental area, dopamine, pain, depression, nociception, anhedonia, electrical brain stimulation

## Abstract

Chronic pain and depression are highly prevalent pathologies and cause a major socioeconomic burden to society. Chronic pain affects the emotional state of the individuals suffering from it, while depression worsens the prognosis of chronic pain patients and may diminish the effectiveness of pain treatments. There is a high comorbidity rate between both pathologies, which might share overlapping mechanisms. This review explores the evidence pinpointing a role for the ventral tegmental area (VTA) as a hub where both pain and emotional processing might converge. In addition, the feasibility of using the VTA as a possible therapeutic target is discussed. The role of the VTA, and the dopaminergic system in general, is highly studied in mood disorders, especially in deficits in reward-processing and motivation. Conversely, the VTA is less regarded where it concerns the study of central mechanisms of pain and its mood-associated consequences. Here, we first outline the brain circuits involving central processing of pain and mood disorders, focusing on the often-understudied role of the dopaminergic system and the VTA. Next, we highlight the state-of-the-art findings supporting the emergence of the VTA as a link where both pathways converge. Thus, we envision a promising part for the VTA as a putative target for innovative therapeutic approaches to treat chronic pain and its effects on mood. Finally, we emphasize the urge to develop and use animal models where both pain and depression-like symptoms are considered in conjunction.

## Introduction

Chronic pain, with a prevalence that ranges from 10% to 40% worldwide, is a major concern in public health. It is the main reason for seeking medical care and represents a major societal burden due to its high socioeconomic impact (Goldberg and McGee, 2011; Breivik et al., 2013; Cohen and Mao, 2014; Cohen et al., 2021; De Ridder et al., 2021). Chronic pain significantly affects the quality of life and psychological wellbeing of patients. It affects not only the physical condition but also the emotional and psychological state of patients, disturbing mood, sleep, or cognitive processes (Baliki et al., 2006; Timmers et al., 2019). Neuropsychiatric disorders (e.g., depression) are often comorbid to chronic pain (Bassols et al., 1999; Mäntyselkä et al., 2001; Frießem et al., 2009; Goldberg and McGee, 2011; Cherif et al., 2020; Cohen et al., 2021). Several studies support the idea that persistent pain increases the likelihood of depression, together with exacerbating its symptoms (Humo et al., 2019). In addition, there are other studies showing fewer functional benefits for antidepressants in patients with comorbid chronic pain (Roughan et al., 2021). Similarly, depression worsens the prognosis of chronic pain patients and diminishes the effectiveness of analgesic treatments (Fishbain et al., 1997; Ren et al., 2016; Sheng et al., 2017). Altogether, it appears probable that there is an association between chronic pain and depression and that their treatment requires a comprehensive approach addressing both the physical and psychological aspects of both conditions.

Chronic pain and depression might share common or overlapping mechanisms. From a pharmacological point of view, antidepressant drugs can be used as analgesics, especially in neuropathic pain and other types of pain that are not well managed with first in line analgesic drugs (e.g., NSAIDs, opioids) (Bonilla-Jaime et al., 2022). Some of these effects may be explained by the activation of the descending pain modulatory system, which consists of neuronal projections from midbrain areas, including the periaqueductal gray (PAG) and the rostral ventral medulla (RVM), to the spinal cord (Obata, 2017). These projections are primarily formed by monoaminergic neurons (releasing noradrenaline and serotonin) that also release endogenous opioid peptides (Hokfelt et al., 1977). Nevertheless, the processing of pain is a complex mechanism and involves other areas, such as some thalamic nuclei, especially in the mediodorsal thalamus, which receive and process sensory information from the periphery (Willis and Westlund, 1997), and in addition have the highest density of opioid receptors (Tempel and Zukin, 1987; Ko et al., 2003). Other relevant areas are the amygdala or the insula, which are involved in the processing of emotional responses to pain (Corder et al., 2019; Takahashi et al., 2019).

Similarly, some analgesics have been proposed as possible antidepressants based on 1) the presence of endogenous opioid peptides in brain areas playing a major role in affective disorders (Jelen et al., 2022), and 2) the affinity for opioid receptors exhibited by some antidepressant drugs (Berrocoso et al., 2009; McHugh and Kelly, 2018). The recent development and approval of the well-established anesthetic and analgesic drug ketamine as a novel antidepressant is another example sustaining that pain and depression may share overlapping mechanisms. While the anesthetic effects of ketamine (and esketamine) are parsimoniously explained by its action as a glutamate N-methyl-D-aspartate receptor (NMDAR) non-competitive antagonist, the molecular mechanisms for its antidepressant effects are more controverted (Zanos et al., 2018). Indeed, we and others have shown that some of the rewarding and antidepressant effects of these drugs are mediated via opioid receptors (Bonaventura et al., 2021; Levinstein et al., 2023).

Taken from a neurochemical perspective, the interplay between the opioidergic and monoaminergic systems and the crossover effects for some analgesic and antidepressant drugs may explain the bidirectional connection between chronic pain and depression. However, from a circuit perspective, other neurotransmitter systems and brain areas might also be involved. One of these areas is the ventral tegmental area (VTA). The VTA is a midbrain region critical for motivation and reward via the projection of dopaminergic neurons to the nucleus accumbens (NAcc) and the prefrontal cortex (PFC) (Koob, 2009; Russo and Nestler, 2013; Cai and Tong, 2022), However, the VTA also projects to other nuclei including the anterior cingulate cortex (ACC), the olfactory bulb, the amygdala, and the hippocampus (Nair-Roberts et al., 2008; Sesack and Grace, 2010; Qi et al., 2016; Montardy et al., 2019). Accordingly, it could play a role both in the processing of pain and emotion. Here, we will review clinical and preclinical evidence supporting the role of the VTA as a possible hub where both pain and emotional processing converge.

## Involvement of the VTA in pain processing

The perception of pain is a highly coordinated and dynamic process that involves interactions between multiple brain areas. As briefly discussed above, some thalamic nuclei are major hubs in the processing of pain (Willis and Westlund, 1997), since they receive information from the periphery and filter and direct the signal to other areas for further processing (Basbaum et al., 2009). The somatosensory cortex plays a crucial role in localizing where pain occurs, while the ACC and the PFC are responsible for processing its emotional and cognitive aspects and for other higher-order cognitive functions, such as decision-making. Other important areas involved in this process are the insula, which integrates sensory, emotional, and cognitive information related to pain; the periaqueductal gray (PAG), modulating the descending pain modulatory system; the hypothalamus, involved in autonomic nervous system responses; and the amygdala, which is part of the limbic system and is mainly associated with its emotional processing (Wiech et al., 2008; Basbaum et al., 2009; Garland, 2012; Moradi et al., 2015; Corder et al., 2019; Huang et al., 2022; Ma et al., 2023).

**FIGURE 1:**
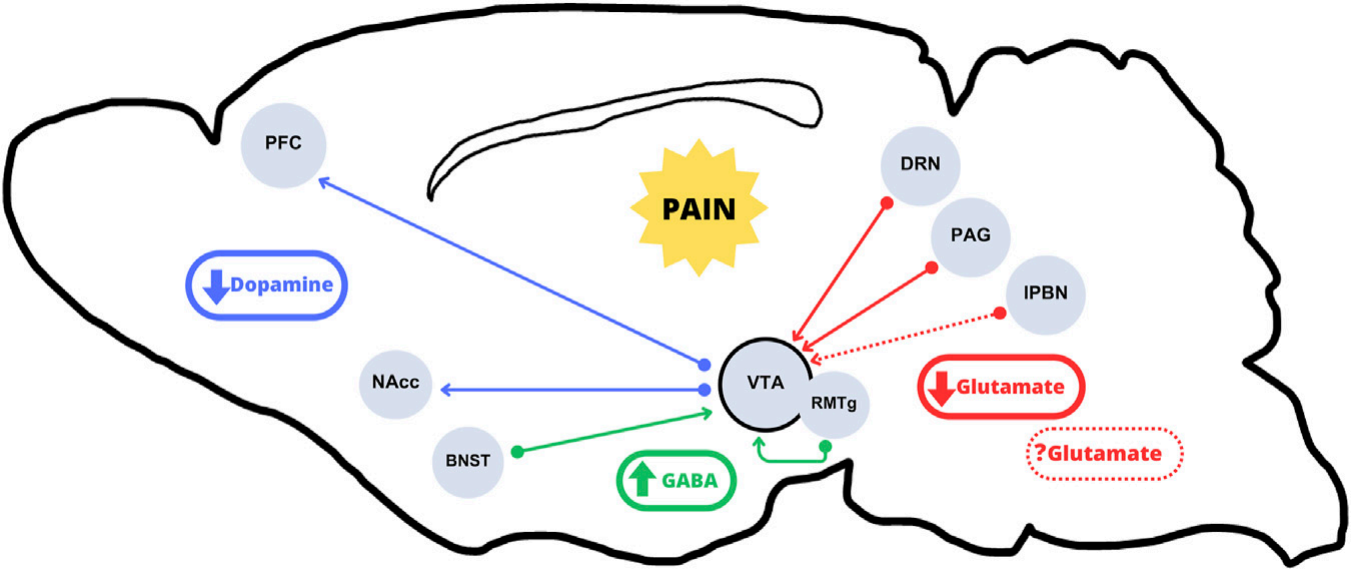
Incoming and arising pathways to and from the VTA subject to alterations under chronic pain conditions. The literature mainly suggests an overall hypoactivity of the VTA dopaminergic outputs in the mesolimbic and mesocortical pathways (blue arrows) in chronic pain conditions. Regarding VTA inputs, there would be a decrease in excitatory glutamate release from projections originating in the DRN and the PAG (solid red arrows). The role of lPBN excitatory neurons, which have been shown to be either activated or inhibited (dotted red arrows), is not clear. The resulting hypoglutamatergia, together with increased GABA release from the BNST and the RMTg (green arrows) would result in an overall VTA dopaminergic output inhibition.

The VTA is not often considered as a primary area involved in pain processing. However, several studies support a putative role for the VTA in pain perception and processing. For instance, a recent study in rats showed that chronic pain decreases dopaminergic activity due to increased inhibition from the bed nucleus of the stria terminalis (BNST) (Takahashi et al., 2019), a region that mediates aversive experiences (Davis et al., 2010; Minami, 2019) (Figure 1). Conversely, in a rat model of neuropathic pain, VTA neurons showed increased burst firing 2 weeks after peripheral nerve injury, which suggested that the increased dopaminergic activity could be an early result of chronic maladaptation to persistent pain (Sagheddu et al., 2015). Of note, these dopaminergic neurons in the VTA are modulated by GABAergic neurons projected from the rostromedial tegmental nucleus (RMTg). These inhibitory projections have been shown to reduce VTA excitability during inflammatory pain (Markovic et al., 2021). Similarly, optogenetic stimulation of VTA reversed allodynia caused by nerve injury in mice (Watanabe et al., 2018). On the other hand, RMTg GABAergic neurons, which express opioid receptors, are thought to mediate the inhibitory effects of opioid drugs in regulating the VTA dopaminergic neurons (Johnson and North, 1992; Fields and Margolis, 2015; Taylor et al., 2019). Indeed, it has been shown that morphine elicits some of its antinociceptive effects through RMTg neurons, thus supporting a critical role of VTA activity not only on the rewarding effects of opioids but also on opioid-mediated analgesia (Taylor et al., 2019).

The activity in the VTA can lead to multifaceted, even contrasting, effects in its projection areas (Lammel et al., 2014) and therefore, the outcomes of its activation are highly context dependent (Belujon and Grace, 2015). The mesolimbic dopaminergic circuit, which consists of the projection of dopaminergic neurons from the VTA to the NAcc, has been shown to be altered in chronic pain conditions (Ren et al., 2016; Campos-Jurado et al., 2019; Yang et al., 2020; Wang et al., 2023). These alterations often involve changes in neurochemistry or neurotransmitter receptors of both the dopaminergic and opioidergic systems [for review see (Taylor et al., 2016; Lin et al., 2023)]. For instance, in different neuropathic pain models, it was observed that an increase in the activity of the VTA dopaminergic neurons had positive effects on pain regulation and its associated mood effects (Abdul et al., 2022; Huang et al., 2022). Similarly, a recent study showed diminished firing and intrinsic excitability of VTA dopaminergic neurons under chronic pain conditions that was due to reduced glutamatergic input from the dorsal raphe nucleus, and that optogenetic modulation of this pathway produced analgesic effects (Wang et al., 2023). Nevertheless, few reports have focused on assessing whether modulating the reward system can exert analgesic effects. For instance, in neuropathic or cancer pain, activation of dopamine neurons projecting to the NAcc restore the allodynia (Watanabe et al., 2018). Similarly, microinjecting dopamine receptor ligands into the NAcc led to reduced injury-induced thermal allodynia (Sato et al., 2022). Particularly, they showed that the specific facilitation of dopamine D1 receptor-expressing medium spiny neurons (MSN) in the NAcc projecting to the VTA led to pain relief, while the inhibition of dopamine D2 receptor-expressing MSNs led to significant antinociceptive effects (Sato et al., 2022). Interestingly, the increased excitability of the D2-containing indirect pathway MSN of the medial shell of the NAcc worsens tactile allodynia following peripheral nerve injury (Ren et al., 2016). Finally, it has been also shown that, in chronic pain conditions, extracellular dopamine release evoked by morphine or cocaine administration is decreased suggesting that a hyporeactive state of the mesolimbic dopaminergic system under chronic pain conditions (Taylor et al., 2015).

Similarly, VTA projections to the PFC are altered upon chronic pain conditions. An example of this, and of the diverse effects of opioid drugs within the brain, is shown in the work by Zhao et al. (2007), in which the injection of morphine in the PFC alleviated mechanical allodynia in a mouse model of neuropathic pain. Another study in mice demonstrated that not necessarily opioid-mediated effects but just increased dopaminergic activity in the PFC, elicited by selective activation of VTA’s dopaminergic neurons, reduced mechanical hypersensitivity (Huang et al., 2020). This effect was explained by an increased PFC-mediated activity in the PAG (Huang et al., 2020). In fact, by using optogenetic manipulations, they demonstrated that the PFC-PAG circuit altered pain behavior by reducing the descending noradrenergic and serotoninergic modulation of spinal pain signals (Huang et al., 2019). The capacity of the PFC to receive nociceptive inputs but also to exert control over the pain sensation can be found elsewhere [for example, see (Apkarian et al., 2005; Ong et al., 2019; Kummer et al., 2020)]. Therefore, there is compelling evidence to support that areas other than the thalamus and canonical pain-related areas play a role in the processing of pain. Another area that might suffer alterations during chronic pain is the claustrum (Atilgan et al., 2022; Ntamati et al., 2023), a multimodal node with brain-wide connectivity and involved in several networks (Atilgan et al., 2022). Interestingly, the claustrum also receives dopaminergic inputs from the VTA (Wong et al., 2021), with dopamine causing a mostly inhibitory response of this structure (Wong et al., 2021), although the precise mechanisms of this inhibition are not yet well understood. However, it is interesting to note that an hypodopaminergic state could explain the reduced excitatory drive of the claustrum onto cortical structures like the ACC (Ntamati et al., 2023). In summary, speculatively, the VTA might have a central role as a flow regulator of the information integrated and processed in areas such as the NAcc, PFC, claustrum, thalamus and PAG. Altogether, these results suggest that the effects elicited by chronic pain through the limbic system could also be viewed as a promising target to reach effective analgesia.

## The VTA as an emerging therapeutic target to treat depression

Depression is a complex, multifaceted, and disabling disorder affecting an estimate of 5% of the world’s adult population (World Health Organization, 2023). Decades of evidence showed the link between depression and the monoaminergic systems, especially the serotoninergic and noradrenergic (Coppen, 1967; Yohn et al., 2017). Although highly controversial and failing to reconcile the diverse symptomatology of depression (see Moncrieff et al., 2022) and its associated correspondence, the serotonergic hypothesis of depression supports the principal therapeutic treatments. The first-line class of antidepressant drugs are selective serotonin re-uptake inhibitors, which increase extracellular serotonin concentrations by blocking presynaptic serotonin transporters. Similarly, other popular antidepressant drug classes include both serotonin-noradrenaline reuptake inhibitors, tricyclic antidepressants, or monoamine-oxidase inhibitors (Cipriani et al., 2018; Lin et al., 2023), which also lead to increased extracellular levels of monoamines. However, all these medications have a delayed onset of action, and around 30%–40% of patients do not show an adequate response (Rush et al., 2006). More recently, psychedelic drugs, which may also act through the serotonergic system but targeting serotonin receptors instead of serotonin re-uptake or degradation, have been proposed as novel treatments for depression (Carhart-Harris et al., 2016; Griffiths et al., 2016; Reiff et al., 2020; Carhart-Harris et al., 2021; Daws et al., 2022). Of note, while their efficacy and safety are still a matter of debate, the role of the serotonergic system in their antidepressant action is also questioned (Hesselgrave et al., 2021; Moncrieff et al., 2022; Moliner et al., 2023).

Despite being the most studied, serotonin and noradrenaline are not the only monoamines implicated in depression. Several studies have shown that changes in the dopaminergic system are associated with both the pathogenesis and treatment of depression (Pani et al., 2000; Nestler and Carlezon, 2006; Yadid and Friedman, 2008; Tye et al., 2013; Belujon and Grace, 2017). Positron emission tomography (PET) imaging studies in humans reported a decrease in dopamine transporter binding potential in depressed patients, which is usually associated with an hypodopaminergic state (Meyer et al., 2001). Accordingly, pharmacological depletions in dopamine led to an increase of depressive symptoms in depressed patients or in subjects with a family history of depression but not in healthy subjects (Ruhé et al., 2007; Hasler et al., 2008). On the other hand, dopamine agonists such as the Parkinson’s disease (PD) medications pramipexole or aripiprazole have antidepressant properties in PD patients with concurrent depression or anhedonia (Lemke et al., 2006).

As a complex disorder, it is not possible to pinpoint a unique brain region or circuit responsible for all the symptoms of depression (Kempton et al., 2011; Spellman and Liston, 2020). Despite this, there are converging reports that observed alterations of the limbic corticostriatal circuitry (precisely, several subregions of the PFC and the NAcc) in anhedonia and reward processing in patients suffering from depression (Johnstone et al., 2007; Siegle et al., 2007; Pizzagalli et al., 2009; Mayberg et al., 1999). The activity of the PFC and the NAcc is heavily modulated by dopaminergic transmission originating in the VTA (Koob, 2009; Russo and Nestler, 2013). Hence, the putative role of the VTA as a therapeutic target for depression has been studied in several preclinical studies in rodents. Interestingly, it has been shown that optogenetic or electrical stimulation of the VTA to NAcc or PFC pathways can ameliorate depressive-like symptoms in animal models of depression (Gersner et al., 2010; Sesia et al., 2010; van der Plasse et al., 2012; Chaudhury et al., 2013; Tye et al., 2013; Bambico et al., 2015; Ferenczi et al., 2016). On the other hand, non-pharmacological treatments of depression (Schlaepfer et al., 2013; Bewernick et al., 2017), such as deep brain stimulation (DBS) or transcranial magnetic stimulation, have also successfully targeted the VTA or the medial forebrain bundle (the main pathway of fibers connecting the limbic midbrain and forebrain) to achieve antidepressant activity in treatment-resistant depressed patients (Schlaepfer et al., 2013; Fenoy et al., 2016; Bewernick et al., 2017). Similar results have been observed with peripheral stimulation: vagus nerve stimulation (VNS) has shown promising results in ameliorating depression scores in clinical trials with patients with mild or moderate symptoms (Fang et al., 2016; Rong et al., 2016). Interestingly, the mechanistic studies of VNS [reviewed in (Carron et al., 2023)] support the idea that its therapeutic effects are due to increased catecholamine release.

Lastly, it is important to highlight that emerging antidepressant therapies, distinct from those directly linked to monoaminergic function -such as ketamine- consistently pinpoint the PFC-NAcc-VTA pathways as crucial anatomical sites of their effects. (Kokkinou et al., 2018; Wu et al., 2021).

## Converging mechanisms between pain and depression in the VTA

Taking the above into consideration, it becomes evident that the VTA is a key structure where both pathogenic and therapeutic mechanisms of pain and depression might converge. Therefore, the VTA might arise as a candidate to target the concurrence of both pathologies. However, despite the high comorbidity between both pathologies in humans (Goldberg and McGee, 2011; Cherif et al., 2020; Cohen et al., 2021) and the extensive clinical data that relate them (Lampl et al., 2016; Roughan et al., 2021; Zhou et al., 2021; Voute et al., 2023), only a few preclinical studies have addressed them together. Of note, these studies generally coincide in suggesting that afferents from the PAG or the parabrachial nucleus (PBN), the dorsal raphe nucleus (DRN) or the BNST directly or indirectly control dopaminergic activity in the VTA efferent regions, such as the NAcc and PFC (Waung et al., 2019; Yang et al., 2021; Zhang et al., 2021; Lee et al., 2023). Hence, the VTA seems to be critical in translating painful stimuli into aversive or depressive-like behaviors.

The work by Waung et al. (2019) demonstrated that excitatory neurons from the ventrolateral PAG (vlPAG) mainly project to VTA GABAergic neurons; however, it is important to highlight that they also detected both excitatory and inhibitory vlPAG inputs into a few dopaminergic neurons (Waung et al., 2019). Similarly, other studies described a parallel glutamatergic input from the lateral PBN (lPBN) to dopaminergic VTA neurons (Yang et al., 2021; Zhang et al., 2021). Therefore, VTA dopaminergic neurons may be relevant for relaying nociceptive signals from the spinal cord to midbrain nuclei. Indeed, when silencing these neurons pain sensation can be blocked. Thus, the ablation of lPBN to substantia nigra pars reticulata glutamatergic neurons was enough to reduce pain-mediated inhibition of dopamine release in mice (Coizet et al., 2010; Yang et al., 2021). Interestingly, one of the reports that addressed chronic pain and depression together (Lee et al., 2023) used a spinal nerve ligation (SNL) mouse model. This model resulted in a dysregulation of the glutamatergic transmission from PAG into the VTA. Then, by using chemogenetic tools to selectively recover the activity of the PAG-VTA pathway, they attenuated the SNL-induced depressive behavior of mice. Altogether, these studies showed that, in mice, pain reduced glutamatergic transmission to the VTA causing a decrease in dopamine release in its efferent areas (NAcc and PFC). The same hypodopaminergic state has been observed in chronic pain conditions, which might be due to the dampening of glutamatergic transmission from the DRN to the VTA, consequently decreasing dopamine release in the NAcc (Wang et al., 2023) (Figure 1). Accordingly, the activation of the DRN-VTA-NAcc pathway would be enough to decrease pain-like hypersensitivity and the concomitant anhedonic state. Another critical area could be the BNST. Several studies (Takahashi et al., 2019; Hara et al., 2020) have revealed the capacity of BNST to modulate the mesolimbic system, not only under chronic pain conditions but also in a depression model. In both cases (pain and depression), it seems likely that the inhibitory inputs to the VTA-projecting BNST neurons would led to neuroplastic changes, which would be a common mechanism between the two diseases (Takahashi et al., 2019; Hara et al., 2020).

As mentioned above, the opioidergic system plays an important role in controlling VTA-mediated dopamine release to the projecting areas (i.e., NAcc), an effect that is critical in regulating opioid reward and reinforcement. Nevertheless, some studies have shown that pain conditions can induce presynaptic MOR desensitization in the VTA, causing an increment in GABA release from RMTg neurons, and resulting in reduced dopamine release to dopaminergic projecting areas (Ozaki et al., 2002; Hipólito et al., 2015; Campos-Jurado et al., 2019). Similarly, chemogenetic activation of the NAcc-projecting VTA dopamine neurons allowed to overcome the pain-reduced motivated behaviors (Markovic et al., 2021). Altogether, pain-induced dysregulation of this circuit affects motivation and reward, supporting that it may act as a trigger to anhedonic-like behaviors. Additionally, apart from the neurochemical changes and the balance shift that leads to a hypodopaminergic state (Figure 1), chronic pain may cause other maladaptive effects in the VTA. For instance, chronic pain induced microglial activation in the VTA, which disrupted the homeostasis in GABAergic neurons and contributed to the decreased extracellular dopamine in the NAcc (Taylor et al., 2015). In conclusion, there are a number of studies supporting the idea that a hypodopaminergic state occurs upon chronic pain conditions, and that the decline in dopamine levels impairs motivated behavior (summarized in Figure 1).

On the other hand, other authors have proposed an alternative scenario. Thus, the development of depression-like behaviors in an animal model of chronic pain correlated with increased firing of the VTA dopaminergic neurons (Zhang et al., 2021). In this study, it was observed that blocking glutamatergic lPBN input to VTA dopamine neurons reversed the depressive-like behavior associated with chronic pain, however it did not affect the induced neuropathic pain sensitivity. In the opposite way, activation of this same circuit in naïve animals resulted in increased depressive-like behaviors, suggesting that the plastic changes induced by pain led to an increased firing neuronal rate that would be responsible for the comorbid emotional impaired state (Zhang et al., 2021).

Taken together, the evidence presented above suggests that the brain pathways incoming and arising from the VTA are part of a key, albeit understudied, circuit to explain the relationship between pain and depression. This hypothesis is further supported by the fact that pharmacological treatments for both pathologies not only overlap but they also directly or indirectly target the VTA. A paradigmatic example of this idea is represented by opioid drugs, which are used to treat pain but have ample actions on mood and have been proposed to treat depression (Browne et al., 2020). However, the abuse liability of opioid drugs makes them far from ideal to treat depression due to the higher vulnerability and comorbidity of addiction in patients with depressive disorders (Swendsen and Merikangas, 2000; Quello et al., 2005; McGrath et al., 2020). Alternatively, non-canonical drugs that target opioid receptors such as ketamine or methadone, which in addition show reduced abuse liability (Cai et al., 2019; Bonaventura et al., 2021), have become depression and pain medications.

Finally, as above-mentioned, a different, non-pharmacological, therapeutic approach to tackle neuropsychiatric disorders is electrical brain stimulation. This technique has been used for treating multiple conditions, such as movement disorders (e.g., Parkinson’s disease), epilepsy, pain and psychiatric conditions like addiction, schizophrenia, or depression, focusing on multiple areas (Lozano et al., 2019). For depression, most studies have proposed targeting areas like the lateral habenula, the NAcc, or the ACC [reviewed in (Schlaepfer and Bewernick, 2013)]. More recently, DBS into the VTA and/or the medial forebrain bundle has also emerged as a suitable treatment. Clinical studies have shown rapid and sustained antidepressant effects (Schlaepfer et al., 2013; Fenoy et al., 2018; Coenen et al., 2019), and they also support its safety as a chronic treatment for up to 6 weeks (Thiele et al., 2018). Regarding pain, DBS has been mostly investigated for cluster headache, a highly disabling pain that tremendously affects patients’ quality of life. Recent studies (Akram et al., 2016; Cappon et al., 2019) have demonstrated that this kind of treatment reduces the frequency and severity of migraine. Interestingly, Cappon et al. (2019) assessed the effects of DBS into the VTA in patients with cluster headache. They assessed the effects of the treatment on cognition, mood, behavior, and quality of life. Their findings suggest that the treatment induced a significant decrease in anxiety and better coping with pain, and an improvement in depression albeit it was not statistically significant (Akram et al., 2016; Cappon et al., 2019).

## Conclusion

About a third of patients suffering from chronic pain also present depressive symptoms (Cherif et al., 2020). In addition, patients with concurrent chronic pain and depression show poorer prognosis than those with chronic pain alone (Fishbain et al., 1997). Hence, individuals with comorbid chronic pain and depression could benefit from a comprehensive approach to address both conditions simultaneously and improve their overall wellbeing and quality of life. However, most of the preclinical research is focused on either the development of novel analgesic treatments or understanding depressive-like behaviors per separate. Here, we highlight the importance of studying pain and depression as coexisting pathologies rather than by themselves to learn more about the converging pathways and mechanisms that can explain their comorbidity and find new effective strategies to treat them in conjunction.

A potential approach could involve targeting, through either pharmacological means or non-pharmacological methods like several forms of brain stimulation, toward brain regions that function as a communication hub connecting both conditions. From the evidence presented in this review it becomes evident that one of such brain regions could be the VTA. Thus, we propose that modulating the activity of the VTA can be regarded as a novel therapeutic opportunity to treat the concurrence of pain and depression. Although further studies will be required to elucidate the precise actions needed to target the VTA with the desirable therapeutic effects, the growing range of neuromodulation technologies, which allow precise and cell-specific control of neural activity, present unprecedented possibilities to tackle these devastating disorders from a newer perspective.
